# Urinary extracellular vesicle-derived miR-126–3p predicts lymph node invasion in patients with high-risk prostate cancer

**DOI:** 10.21203/rs.3.rs-4164213/v1

**Published:** 2024-03-29

**Authors:** Liang Dong, Cong Hu, Zehua Ma, Yiyao Huang, Greg Shelley, Morgan D. Kuczler, Chi-Ju Kim, Kenneth W. Witwer, Evan T. Keller, Sarah R. Amend, Wei Xue, Kenneth J. Pienta

**Affiliations:** Ren Ji Hospital, Shanghai Jiao Tong University School of Medicine; Ren Ji Hospital, Shanghai Jiao Tong University School of Medicine; Ren Ji Hospital, Shanghai Jiao Tong University School of Medicine; Nanfang Hospital Southern Medical University; University of Michigan; The Brady Urological Institute, Johns Hopkins University School of Medicine; The Brady Urological Institute, Johns Hopkins University School of Medicine; Johns Hopkins University School of Medicine; University of Michigan; The Brady Urological Institute, Johns Hopkins University School of Medicine; Ren Ji Hospital, Shanghai Jiao Tong University School of Medicine; The Brady Urological Institute, Johns Hopkins University School of Medicine

**Keywords:** prostate cancer, lymph node invasion, extracellular vesicles, microRNAs

## Abstract

To investigate extracellular vesicles (EVs) biomarkers for predicting lymph node invasion (LNI) in patients with high-risk prostate cancer (HRPCa), plasma and/or urine samples were prospectively collected from 45 patients with prostate cancer (PCa) and five with benign prostatic hyperplasia (BPH). Small RNA sequencing was performed to identify miRNAs in the EVs. All patients with PCa underwent radical prostatectomy and extended pelvic lymph node dissection. Differentially-expressed miRNAs were identified in patients with and without pathologically-verified LNI. The candidate miRNAs were validated in low-risk prostate cancer (LRPCa) and BPH. Four miRNA species (e.g. miR-126–3p) and three miRNA species (e.g. miR-27a-3p) were more abundant in urinary and plasma EVs, respectively, of patients with PCa. None of these miRNA species were shared between urinary and plasma EVs. miR-126–3p was significantly more abundant in patients with HR PCa with LNI than in those without (P = 0.018). miR-126–3p was significantly more abundant in the urinary EVs of patients with HRPCa than in those with LRPCa (P = 0.017) and BPH (P = 0.011). In conclusion, urinary EVs-derived miR-126–3p may serve as a good biomarker for predicting LNI in patients with HRPCa.

## 1. Introduction

Prostate cancer (PCa) is one of the most common malignancies among men worldwide. The number of new cases in 2023 in the USA is estimated to reach 288,300, ranking first among all types of cancer in men, and PCa has the second highest number of estimated deaths[1, 2]. Metastatic PCa remains incurable despite advances in therapeutics in recent decades.^2^ Recent studies have demonstrated that the lymph nodes, rather than the bone marrow, may be the true reservoir of micrometastatic disease in patients with PCa. In mice, lymph node metastases can invade local blood vessels, exit the nodes, and colonize in distant organs[3]. Extended pelvic lymph node dissection (ePLND) is an effective modality for treating and controlling lymph node invasion (LNI). Approximately 20% of patients with high-risk PCa (HRPCa) have LNI[4]. LNI is a major risk factor for PCa recurrence after radical prostatectomy (RP).

Several nomograms based on patients’ clinicopathological features have been used as tools to predict the risk of LNI preoperatively[5–7]. Although these nomograms share a similar predictive ability, an optimal nomogram has yet to be developed[8]. As a result, ePLND is not conducted precisely, resulting in missed identifications of patients with a high risk of developing biochemical recurrence after RP. In addition, approximately 80% of patients who undergo ePLND may be overtreated and suffer from unnecessary complications, such as lymphatic leakage, lymphoedema, and thromboembolism[9]. Therefore, the development of novel diagnostic biomarkers to precisely identify patients with PCa with a high risk of LNI is necessary.

Liquid biopsy is a less invasive method used to trace cancer and has emerged as a candidate to replace invasive tissue biopsy for more frequent and accurate cancer sampling, allowing for precision medicine. Extracellular vesicles (EVs) are particles with a lipid bilayer that are released into the extracellular space by living cells. EVs carry molecular cargo, such as proteins, nucleic acids, and lipids[10]. Studies regarding EVs in basic biology, as therapeutics, and as biomarkers in liquid biopsies have increased. Recent findings suggest that EVs play a crucial role in building premetastatic niches in distant organs, including lymph nodes[11, 12]. Therefore, EV-derived biomarkers for the prediction of LNI must be investigated. In this study, candidate molecules from plasma and urinary EVs that may be useful to predict LNI in patients with HRPCa are identified.

## 2. Materials and Methods

### 2.1 Patients and sample collection

**Figure 1:**
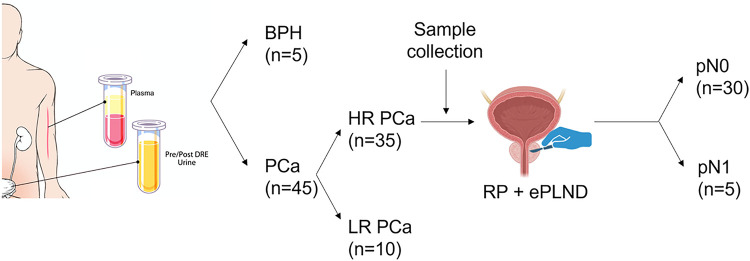
Blood and urine sample collection. Forty-five patients with PCa and five with BPH were included in this study. Abbreviations: BPH, benign prostate hyperplasia; PCa, prostate cancer; HR PCa, high-risk PCa; LR PCa, low-risk PCa; RP, radical prostatectomy.

Five patients with benign prostatic hyperplasia (BPH) and 45 with PCa who were treated at Johns Hopkins Hospital in 2018 were recruited for this study. All diagnoses were based on prostate needle biopsy findings (Fig. 1). Patient age, prostate specific antigen (PSA) level, and Gleason score were recorded. Thirty-five patients were classified with HRPCa and 10 were classified with low-risk PCa (LRPCa) based on the American Urological Association (AUA) guidelines. The patients with HRPCa underwent RP and ePLND. Postoperative pathology was used to identify patients with pN0 (n = 30) and pN1 (n = 5). Plasma and urine samples were collected preoperatively. The plasma and urine samples were prepared as previously described[13].

### 2.2 Separation of EVs

EV original 70-nm columns (IZON Science, Cambridge, MA, USA) were used to separate the EVs as previously described[13]. Urine (40 mL) and plasma (2 mL) was concentrated to 0.5 mL using 10 kDa molecular weight cut off (MWCO) filters (Centricon^®^ Plus-70 Centrifugal Filters and 10 kDa MWCO Amicon^®^ Ultra-2 Centrifugal Filters, MilliporeSigma, Burlington, MA, USA). The columns were washed with phosphate-buffered saline (PBS). Samples (≤ 0.5 mL) were loaded onto the column. Fourteen sequential 0.5-mL fractions were eluted using PBS. The EV-enriched fractions (EVEF) were fractions 7–10. The EVEF were pooled and concentrated using the kDa centrifugal filters to a final volume of 100 μL.

### 2.3 Transmission electron microscopy

The morphology and size of the isolated EVs were visualized using transmission electron microscopy (TEM). First, ultra-thin carbon coated 400 mesh copper grids were glow-discharged (EMS GloQube, USA) for better adsorption of the EVs. Each 10-μL sample of EVs was dropped onto the grid and adsorbed for five minutes before blotting and negative staining with 1% uranyl acetate and methylcellulose. After the grids were dried in the dark, they were visualized using TEM (HT7800, Hitachi, Japan) at 100kV.

### 2.4 Western blotting

Western blotting was performed to assess the expression of EV and non-EV protein markers. After boiling at 95°C for 10 min, equal amounts of proteins from EVs were placed on 12% SDS Mini-PROTEAN^®^ TGX Stain-Free^™^ protein gels (Bio-Rad Laboratories, USA). Nitrocellulose membranes (Trans-Blot^®^ Turbo^™^ Mini Nitrocellulose, Bio-Rad Laboratories) were incubated overnight at 4°C with the following antibodies: Flot-1 (ab133497, Abcam, UK); CD63 (10628D, Thermo Fisher Scientific, USA); CD81 (sc-7637, Santa Cruz Biotechnology, USA) and Calnexin (ab22595, Abcam). After the washing procedure with tris-buffered saline and tween 20, the nitrocellulose membranes were incubated with IRDye^®^ 680RD goat anti-mouse IgG (92668070, LI-COR Biosciences, USA) and IRDye^®^ 800CW goat anti-rabbit IgG (92632211, LI-COR Biosciences, USA) for 1 h at room temperature. Blots were imaged using an Odyssey^®^ 9120 Infrared Imaging System (LI-COR Biosciences, USA).

### 2.5 Nano-flow cytometry

The particle size distribution and concentrations of the EVs were analyzed using nano-flow cytometry (nFCM). All preparations were performed according to the manufacturer’s instructions. In each test, all particles that passed through the detector for 1 min were recorded. Particle number concentration and size distribution were analyzed after calibration using 250-nm polystyrene beads (for concentration) and a Silica Nanosphere Cocktail (S16M-Exo, NanoFCM, Inc., China; for sizing). The flow rate and side scattering intensity were converted into corresponding particle concentrations and size distributions using nFCM software (NanoFCM Profession V2.0, NanoFCM, Inc., China).

### 2.6 RNA isolation and quality control

Total RNA was extracted from the samples using TRIzol (Invitrogen, USA). The RNA purity was checked using the kaiaoK5500^®^Spectrophotometer (Kaiao, China). RNA integrity and concentration were assessed using an RNA Nano 6000 Assay Kit on a Bioanalyzer 2100 system (Agilent Technologies, USA).

### 2.7 Small RNA sequencing

Small RNA libraries were constructed from 8 μL of RNA extracted from plasma and urine EVs using the D-Plex Small RNA-seq Kit for Illumina (C05030001, Diagenode, Belgium). Indexes were attached using D-Plex 24 Single Indexes for Illumina (C05030010, Diagenode, Belgium) according to the manufacturer’s protocol. The yield and size distribution of the small RNA libraries were assessed using a Fragment Analyzer instrument with a DNA 1000 chip (5067–1504, Agilent Technologies, USA). After library size selection from 120–200 bp using BluePippin (HTG3010, 3% agarose, Sage Science, USA), multiplexed libraries were equally pooled to 1 nM for 151-bp paired-end sequencing on the NovaSeq 6000 system (Illumina, USA). Bcl2fastq2 Conversion Software (Illumina, USA) was used to generate de-multiplexed Fastq files.

### 2.8 RNA sequencing data analyses

The original BAM files were converted into FASTQ format using Picard tools (SamToFastq command). The polyA-tails and the 5’-UMI sequences were trimmed from the raw reads using cutadapt software (http://code.google.com/p/cutadapt/). Trimmed and size-selected (> 15 nt) reads were sequentially aligned to hg38 reference transcriptomes using Bowtie software (http://bowtie-bio.sourceforge.net) with a mismatch tolerance of zero. All reads were mapped to RNA species with low sequence complexity and/or a high number of repeats: rRNA, tRNA, RN7S, snRNA, snoRNA/scaRNA, vault RNA, RNY, and mitochondrial chromosome (mtRNA). All reads that did not map to the above RNAs were sequentially ligned to mature miRNAs, pre-miRNAs, protein-coding mRNA transcripts (mRNA), and long non-coding RNAs (lncRNAs). Reads that did not map to the above RNAs were aligned with the remaining transcriptomes. Finally, all reads that did not map to the human transcriptome were aligned to the human reference genome (rest hg38), which corresponded to the introns and intergenic regions. Differential miRNA expression was quantified (P < 0.05) using the R/Bioconductor package DESeq2, as previously described[14]. miRNAs were screened for differential abundance based on a foldchange ≥ 2 and a P value < 0.05.

### 2.9 Database analysis

The RNA-Seq expression data of 51 normal prostate tissues and 490 PCa tumors from The Cancer Genome Atlas (TCGA; https://portal.gdc.cancer.gov/) database were used to validate the EV findings.

### 2.10 Statistical analysis

Student t-test and chi-square test were used to estimate the difference of differentially expressed miRNAs in groups. When data did not accord with normal distribution, Mann-Whitney U test was adopted. P < 0.05 was considered as statistically significant. GraphPad Prism 9.0 (USA) was used for graph generation.

## 3. Results

### 3.1 Patient characteristics

The mean ages of patients with HRPCa, LRPCa, and BPH were 68.34 years (range: 51–89 years), 62.25 years (range: 50–78 years) and 58.78 years (range:42–85 years), respectively (Table 1). In the HRPCa group, one patient (2.9%) had a Gleason score of 7 at the time of prostatectomy, 16 patients (45.7%) had a score of 8, and 18 patients (51.4%) had a score of 9. All ten patients with LRPCa had a Gleason score of 6 at the time of biopsy. Twenty-six patients (74.3%) with HRPCa had cT1–cT2a lesions, 6 (17.1%) had cT2b–cT3a lesions, and 3 (8.6%) had ≥ cT3b lesions. All ten patients with LRPCa had cT1 lesions. The mean baseline PSA values were 17.870 ng/ml (range: 4.4–152 ng/ml), 5.581 ng/ml (range: 4.0–8.1 ng/ml), and 3.018 ng/ml (range: 1.7–4.0 ng/ml) in the HRPCa, LRPCa, and BPH groups, respectively. Thirty patients (85.7%) with HRPCa had pN0 lesions and five (14.3%) had pN1 lesions. All ten patients with LRPCa had pN0 lesions.

### 3.2 EVs characterization

**Figure 2:**
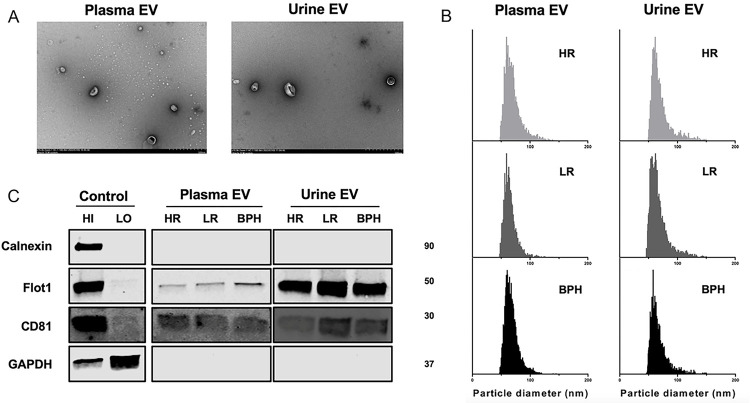
Characterization of separated EVs from plasma and urine. A. TEM confirms the cup-shaped EVs. Scale bar is 500 nm. B. The size distribution and concentrations of separated EVs is analyzed via nFCM. C. EV markers are verified using western blotting. Abbreviations: HI, high abundance control; LO, low abundance control; BPH, benign prostate hyperplasia; HR, high-risk; LR, low-risk.

Based on the Minimal Information for Studies of EVs (MISEV) guidelines[15], we characterized EVs from the urine and plasma of patients. Cup-shaped morphology (a characteristic artifact of fixation) and sizes consistent with EVs were observed by TEM (Fig. 2A). By nFCM, the particle diameter distribution ranged from 30–150 nm (Fig. 2B). EV markers (CD81 and Flot1) were identified on Western blotting, and depletion of a contamination marker (calnexin) was confirmed (Fig. 2C). These characteristics of urine and plasma EVs were not significantly different between patients with HRPCa, LRPCa, and BPH.

### 3.3 Differential EV-derived miRNA abundance in the urine of patients with PCa

**Figure 3:**
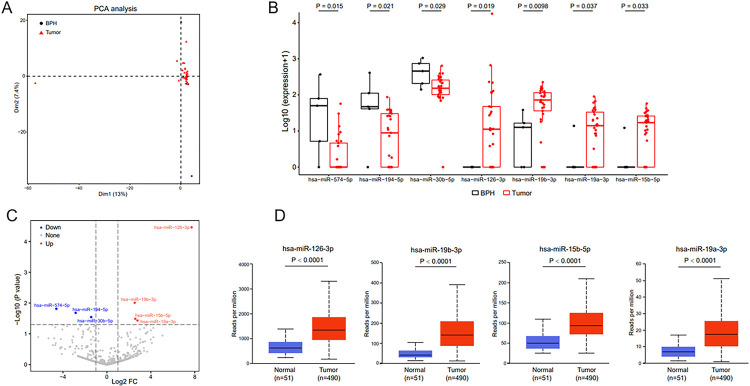
Differential urine EV-derived miRNA expressions in patients with PCa. A. The PCA plot shows clustering of different miRNAs in patients with PCa and BPH. B. Seven miRNAs have different expressions between the BPH and PCa groups. C. The volcano plot shows the seven differential miRNAs between the BPH and PCa groups. D. The TCGA database is used to verify the differential expression of miRNAs. Foldchange ≥ 2 and P < 0.05.

miRNA abundance differed between patients with PCa and BPH (Fig. 3A). miR-574–5p, miR-194–5p, and miR-30b-5p expressions were lower in urinary EVs from patients with PCa than in EVs from the urine of patients with BPH. miR-126–3p, miR-19b-3p, miR-15b-5p, and miR-19a-3p expressions were relatively high (Figs. 3B and 3C). The latter four upregulated miRNAs were subsequently validated in PCa tissues using data from the TCGA database (Fig. 3D).

### 3.4 Differential EV-derived miRNA abundance in the plasma of patients with PCa

**Figure 4:**
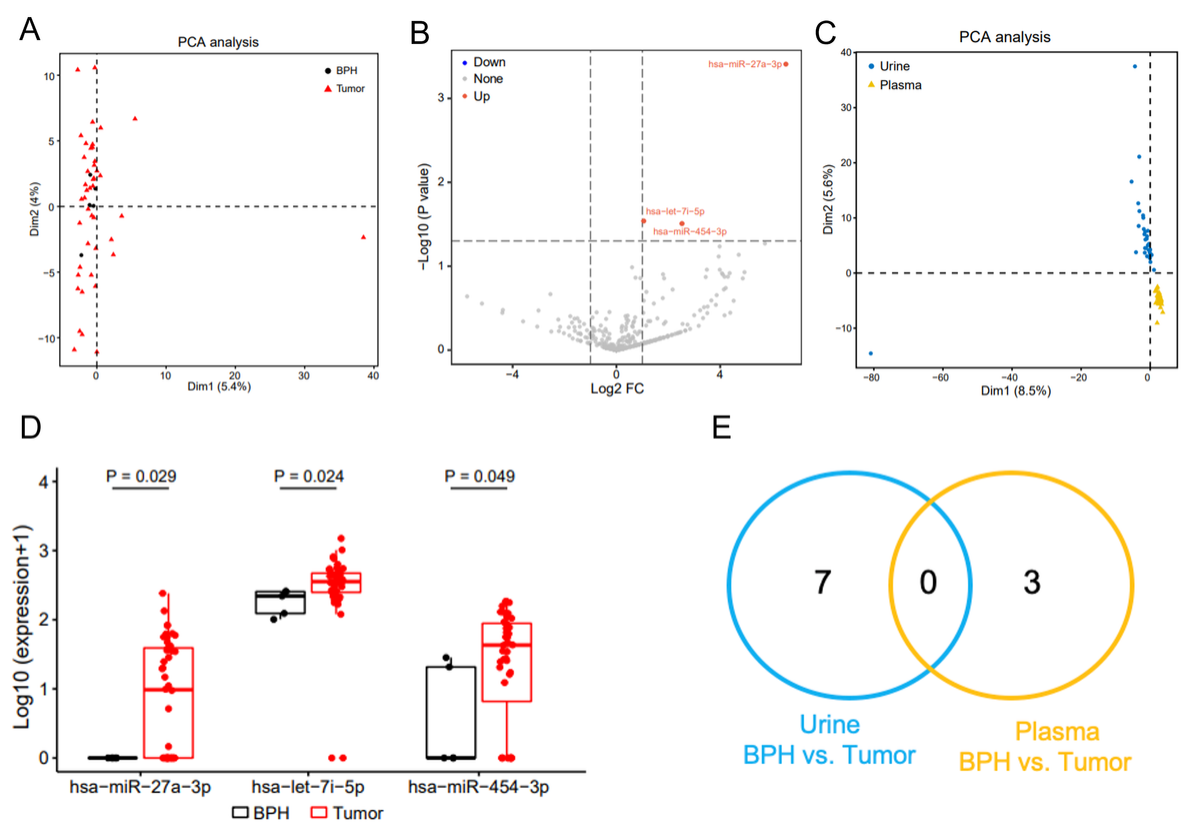
Differential plasma EV-derived miRNA expressions in patients with PCa. A.The PCA plot shows clustering of the different miRNAs in patients with PCa and BPH. B. A volcano plot shows three upregulated miRNAs in the PCa group.C. The PCA plot shows significant differences between plasma and urine EV based on the differential miRNAs in patients with PCa and BPH. D. The differential expressions of three miRNAs between the BPH and PCa groups are shown. E. Venn diagrams present the overlap of dysregulated miRNAs between matched urine- and plasma-derived EVs. Foldchange ≥ 2 and P < 0.05.

The miRNAs in the plasma EV were not significantly different between patients with PCa and those with BPH (Fig. 4A). However, miR-27a-3p, let-7i-5p, and miR-454–3p were more enriched in patients with PCa (P = 0.029, P = 0.024, P = 0.049, respectively) (Figs. 4B and 4D).

Further analysis of whether urine- and plasma-derived EVs contained had the same significantly dysregulated molecules was performed. PCA revealed significant differences between the urine- and plasma-derived EVs from patients with PCa and BPH (Fig. 4C). However, the dysregulated miRNAs did not overlap between the matched urine- and plasma-derived EVs (Fig. 4E).

### 3.5 Upregulated miR-126–3p in urinary EVs helps predict LNI in patients with HRPCa

**Figure 5:**
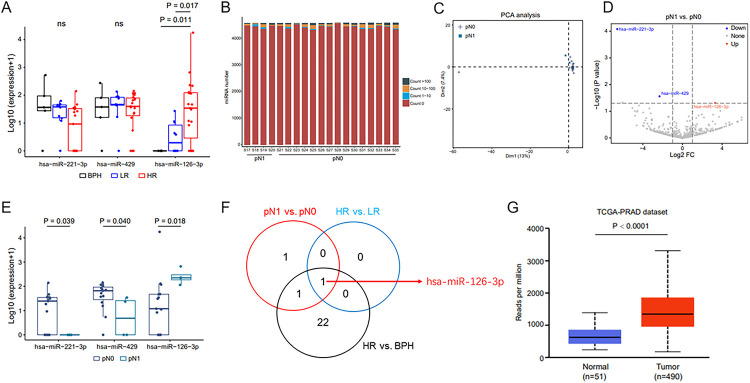
Upregulated miR-126–3p in urine EVs helps predict LNI in patients with HRPCa. A.Urine EV-derived differential miRNA expressions in the BPH, LRPCa, and HRPCa groups are shown. B. The expressions of miRNA in the pN0 and pN1 groups are shown. C. PCA identifies no significant distinctions between the pN0 and pN1 groups. D. A volcano plot demonstrates the differential miRNAs in the pN0 and pN1 groups. E. The differential expressions miRNAs between the pN0 and pN1 groups are shown. F. Venn diagrams present the overlap of dysregulated miRNAs between different comparison groups. G. The TCGA database is used to verify miR-126–3p expression. Foldchange ≥ 2 and P < 0.05. Abbreviations: BPH, benign prostate hyperplasia; PCa, prostate cancer; HR, high-risk PCa; LR, low-risk PCa.

To screen for co-upregulated miRNAs, analyses were performed in patients with HR and LR PCa, in HR patients at the pN0 and pN1 stages, respectively. The levels of miR-221–3p and miR-429 in patients with HRPCa were not significantly different compared with those of patients with BPH or LRPCa. However, the expression of miR-126–3p was proportional to disease severity (P < 0.05) (Fig. 5A). The miRNA levels were not significantly different between patients with HRPCa at stage pN0 and pN1 (Fig. 5B). While PCA revealed that no significant distinctions were found between pN0 with pN1 (Fig. 5C), the expression of miR-221–3p and miR-429 were lower in pN1 group while miR-126–3p was higher than pN0 (Figs. 5D and 5E). Interestingly, after comparing the miRNAs of urinary EVs in the three populations (PCa vs. BPH, HR vs. LR, and pN1 vs. pN0), we found that miR-126–3p expression in the urinary EVs was consistently upregulated in PCa, HR and pN1 groups (Fig. 5F). The expression of miR-126–3p was similarly elevated in PCa tissues (Fig. 5G).

**Figure 6:**
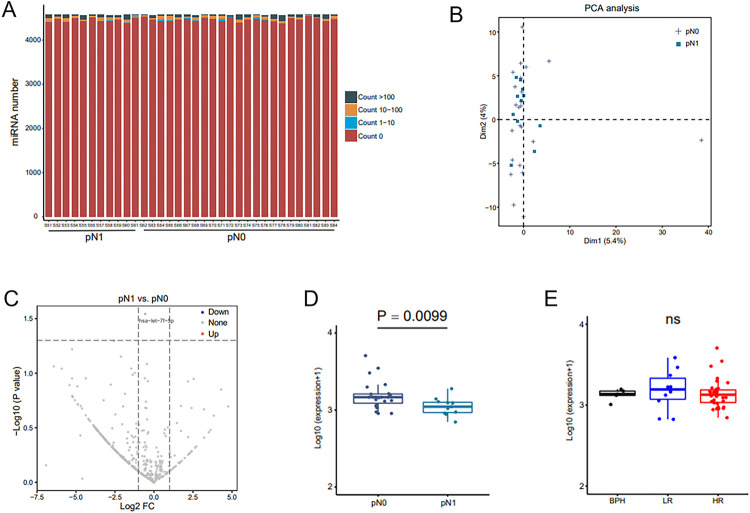
The plasma EV-derived miRNA is not applicable to predict LNI in HRPCa. A. The expression of miRNA in the pN0 and pN1 groups is shown. B. A volcano plot demonstrates the differential miRNAs between the pN0 and pN1 groups. C. A volcano plot demonstrates the differential miRNAs in the pN0 and pN1 groups. D. The differential expressions of miRNAs between the pN0 and pN1 groups are shown. E. The differential expression of miRNA hsa-let-7f-5p in the BPH, LRPCa, and HRPCa groups is shown. Foldchange ≥ 2 and P < 0.05. Abbreviations: BPH, benign prostate hyperplasia; PCa, prostate cancer; HR, high-risk PCa; LR, low-risk PCa.

Based on plasma EVs-derived miRNA abundance, expression profile was also analyzed (Fig. 6A). In pN0 and pN1 group, the differences were not well distinguished (Figs. 6B and 6C). The expression of hsa-let-7f-5p did not differ between the groups (HR vs. LR, pN1 vs. pN0) (Figs. 6D and 6E).

## 4. Discussion

Metastasis is the leading cause of death in patients with PCa. More than 15% of patients with PCa have LNI at the pathological evaluation stage after RP and ePLND[16]. LNI is an important prognostic factor for tumor recurrence in patients with PCa, and patients with pN1 have a worse prognosis than patients with pN0. Tailored treatment based on the appropriate staging should be achieved to maximize the balance between oncological control and adverse effects[17]. The current imaging-based approach to diagnose LNI has limited sensitivity and is not well suited for clinical needs[18, 19].

EVs are distributed in a wide range of body fluids, including blood, urine, and cerebrospinal fluid. They are abundant and have good stability. As a convenient and non-invasive modality, EV-based liquid biopsy techniques play a crucial role in the diagnosis and prognosis of tumors[20]. The identification of key biomarkers of LNI in patients with PCa based on EVs will enable the selection of candidate patients for relevant therapies. As important components of EV, miRNAs are extensively used for the management of patients with PCa[21]. Semen EV-derived miRNA (miR-142–3p, miR-142–5p, and miR-223–3p) and PSA levels can be combined to discriminate PCa from BPH[22]. Urinary miR-532–5p in EVs can help predict the recurrence of PCa[23]. Dysregulated EV-derived miRNAs (such as miR-126–3p and miR-19b-3p) in the urine and plasma may provide new insights regarding the diagnosis of PCa. Plasma samples often contain information from various cellular sources, and may lead to misconstruction. After digital rectal examination, prostate biomarkers are common in the urine. However, the identification of valid biomarkers that can be applied clinically is challenging due to several factors, including the heterogeneity of the population, various sequencing technologies, and different EV isolation methods. To obtain the most efficacious markers by excluding as much extraneous interference as possible, common differentially expressed molecules in matched samples in the PCa and BPH groups were identified. Although no markers reflecting disease severity were common between the urine and plasma, a stable EV-derived miRNA (hsa-miR-126–3p) that was expressed differentially in the HRPCa and LRPCa groups as well as the pN1 and pN0 groups was identified. Qu et al. reported that miR-126–3p improves the proliferation rate and inhibits the apoptosis rate of cells[24]. miR-126–3p promotes tumor angiogenesis, invasion, and migration[25–27]. As an immune-related miRNA, it is a clinically relevant biomarker of disease severity and treatment response[28, 29]. miR-126–3p has been reported as a predictor of LNI in patients with gastric cancer[30]. Another study reported that urine EV-derived miR-126–3p may be a potential marker for the diagnosis of PCa[31].

These findings support the application of miRNAs for predicting LNI in patients with PCa. However, this study is not without limitations. The specific mechanisms underlying PCa metastasis require further exploration. The sample size in this study is small, and more prospective cohorts are needed to validate its efficacy. In addition, the predictive efficacies of other molecules in EVs, such as proteins, were not investigated, and require further research.

## 5. Conclusions

Urinary EV-derived hsa-miR-126–3p is a potential predictor for LNI in patients with PCa; however, no miRNA biomarkers were identified in plasma EVs.

## Figures and Tables

**Table 1.** Patient characteristics.

Characteristic	high-risk PCa (n=35)	low-risk PCa (n=10)	BPH (n=5)

Age, years	68.34	62.25	58.78
Median (SD, range)	(9.01,51–89)	(8.96,50–78)	(9.56,42–85)
pathological N stage n (%)			
pN0	30 (85.7)	10 (100.0)	/
pN1	5 (14.3)		/
PSA, ng/ml Median (range)	17.870 (4.4–152)	5.581 (4.0–8.1)	3.018(1.7–4.0)
Biopsy Gleason Score, n (%)			
< 8	1 (2.9)	10 (100.0)	/
≥ 8	34 (97.1)	0 (0.0)	/
Clinical T stage n (%)			
< T2c	30 (85.7)	10 (100.0)	/
≥ T2c	5 (14.3)	0 (0.0)	/

## Data Availability

The small RNA sequencing data were included in supplementary information.

## Abbreviations

EVs: extracellular vesicles
LNI: lymph node invasion
PCa: prostate cancer
HRPCa: high-risk prostate cancer
BPH: benign prostatic hyperplasia
LRPCa: low-risk prostate cancer
RP: radical prostatectomy
ePLND: extended pelvic lymph node dissection
PSA: prostate specific antigen
TEM: transmission electron microscopy
nFCM: nano-flow cytometry
